# The Role of Microglial Phagocytosis in Ischemic Stroke

**DOI:** 10.3389/fimmu.2021.790201

**Published:** 2022-01-10

**Authors:** Junqiu Jia, Lixuan Yang, Yan Chen, Lili Zheng, Yanting Chen, Yun Xu, Meijuan Zhang

**Affiliations:** ^1^ Department of Neurology, Drum Tower Hospital, Medical School and The State Key Laboratory of Pharmaceutical Biotechnology, Institute of Brain Science, Nanjing University, Nanjing, China; ^2^ Jiangsu Key Laboratory for Molecular Medicine, Medical School of Nanjing University, Nanjing, China; ^3^ Jiangsu Province Stroke Center for Diagnosis and Therapy, Affiliated Drum Tower Hospital, Medical School of Nanjing University, Nanjing, China; ^4^ Nanjing Neuropsychiatry Clinic Medical Center, Affiliated Drum Tower Hospital, Medical School of Nanjing University, Nanjing, China

**Keywords:** phagocytosis, microglia, ischemic stroke, signaling receptors, prognosis

## Abstract

Microglia are the resident immune cells of the central nervous system that exert diverse roles in the pathogenesis of ischemic stroke. During the past decades, microglial polarization and chemotactic properties have been well-studied, whereas less attention has been paid to phagocytic phenotypes of microglia in stroke. Generally, whether phagocytosis mediated by microglia plays a beneficial or detrimental role in stroke remains controversial, which calls for further investigations. Most researchers are in favor of the former proposal currently since efficient clearance of tissue debris promotes tissue reconstruction and neuronal network reorganization in part. Other scholars propose that excessively activated microglia engulf live or stressed neuronal cells, which results in neurological deficits and brain atrophy. Upon ischemia challenge, the microglia infiltrate injured brain tissue and engulf live/dead neurons, myelin debris, apoptotic cell debris, endothelial cells, and leukocytes. Cell phagocytosis is provoked by the exposure of “eat-me” signals or the loss of “don^’^t eat-me” signals. We supposed that microglial phagocytosis could be initiated by the specific “eat-me” signal and its corresponding receptor on the specific cell type under pathological circumstances. In this review, we will summarize phagocytic characterizations of microglia after stroke and the potential receptors responsible for this programmed biological progress. Understanding these questions precisely may help to develop appropriate phagocytic regulatory molecules, which are promoting self-limiting inflammation without damaging functional cells.

Stroke has a great impact on public health and is still the leading cause of death and disability in the world (1). Obstruction of the blood and oxygen supply after ischemic stroke is the initial reason for cell death. The intense changes of circumstances like accumulation of cell debris or infiltration of immune cells in the brain may induce the secondary progression of cell injury (2). Strategies aiming at restoring cerebral perfusion, namely, intravenous rt-PA injection and emergent endovascular intervention have been written in stroke therapeutic guidelines (3, 4). Plenty of stroke patients cannot receive this treatment for missing the time windows although sizable efforts have been made to shorten the door to needle time (DNT). Hence, it is imperative to develop drugs that extend the time window through alleviating the secondary injury and promoting rehabilitation of ischemic stroke.

Microglia, the professional phagocyte of the brain, are capable of engulfing cell debris and pruning synapses in developing brain and various cerebral diseases (5). Despite being less commonly known, a growing body of literature has linked microglial phagocytosis with stroke recovery. In this review, we will summarize phagocytic characterizations of microglia after stroke and the potential receptors responsible for this programmed biological progress.

## Brief Introduction of Microglia Properties After Ischemic Stroke

### Microglia Accumulate in Large Numbers After Ischemic Stroke

Originating from myeloid precursors, microglial cells born in the yolk sac invade the central nervous system (CNS) during early embryonic development, which continuously supervises the brain parenchyma and protects against damage-associated pathogens (6). Upon ischemic stroke, microglia are rapidly activated and accumulate in large numbers at the site of infarction, which is also defined as microgliosis (7). Whether microglia are replenished *in situ* or derived from circulatory precursors is the subject of great interest. Taking advantage of a mouse photothrombosis stroke model, Li et al. reported that reactive microglia increased within minutes and were recruited to the infarcted area continuously during the first week after stroke induction (7). Additionally, local resident microglia division rather than recruitment of circulating macrophage was the main source of microgliosis (7, 8). Researchers then begin to search whether microglia progenitors exist in the brain, which gives rise to the newborn microglia after cerebral injury. It is considered that microglia progenitor cells in the adult brain may be lacking. The rapidly proliferated microglia after the injury are solely derived from residual microglia rather than microglia progenitors (9).

### Functions of Microglia Were Heterogenous After Ischemic Stroke

It is widely accepted that targeting microglia activation could inhibit inflammatory injury and facilitate better stroke recovery (10). However, depletion of microglial cells exacerbates ischemic injury and dysregulates neuronal network activity (11, 12). It is predicted that detrimental outcomes of excessive microglial activation after ischemia could be counterbalanced by beneficial outcomes, namely, phagocytosis and release of trophic factors. It also provides a hint that microglia demonstrate great heterogeneity, which calls for further investigations regarding microglia subtypes or specific functions. Upon the challenge of ischemic stroke, rest microglia can be activated in response to the inflammatory triggers and differentiates into two phenotypes like macrophage: M1 and M2. M1 macrophages (classically activated) are thought to be neurotoxic and are related to the production of pro-inflammatory cytokines, such as interleukin-6 (IL-6) and tumor necrosis factor-α (TNF-α), while M2 macrophages (alternatively activated) are neuroprotective and promote tissue repair and stroke recovery (13). It is worth mentioning that M2 microglia are proven to show the ability of phagocytosis and their capability of cleaning neuronal debris reduces brain damage after stroke (14). *In vitro* assays suggest that IL-10-induced M2 microglia present enhanced phagocytosis abilities (15). However, this classification method could not adequately reflect the complicated microglial characteristics in the activated state. In recent studies, researchers even failed to find pure M1 or M2 state *in vivo* (16). With the development of single cell-sequencing techniques, scientists prefer to designate the microglia profile regarding its unique functions in specific diseases. For instance, one unique microglia population with the potential to restrict neurodegeneration of Alzheimer’s disease is named neurodegenerative microglia (DAM) (17, 18). Therefore, we also expect to characterize microglia in ischemic stroke from a functional perspective.

### Microglia Invade Infarcted Areas Earlier Than Macrophages

Due to similar histological phenotypes, the contribution of activated resident microglia or infiltrated macrophages to stroke pathology has been difficult to distinguish. On Day 1 after focal cerebral ischemia, the microglia amount remains unchanged (19). However, they switch their morphology into an ameboid and rounded shape with acquiring phagocytic properties and phagocytose neuronal cell debris. Thereafter, the microglia continue to proliferate during the first two weeks and show the most powerful phagocytic capacity within the first 2 days of stroke, which proceeds and predominates over phagocytes of hematogenous origin (19). Similarly, histological studies of green fluorescent protein (GFP) transgenic bone marrow chimeric mice with transient or permanent stroke models pointed that blood-derived macrophages infiltrated infarct area with a delay of at least 24 to 48 h after stroke onset (20, 21).

Taken together, studies suggest that macrophages invade the ischemic site of stroke later than microglia. The microglia are the main phagocytes during the first three days after stroke (19–21). In contrast to the rapid microglial response, the macrophages are rarely detected during the first 48 h and then gradually increase with the peak time in the first week after stroke (22). Transcriptomic studies of macrophages at Day 5 post stroke showed that infiltrated macrophages promote efferocytosis and inflammation resolution after ischemic stroke (23).

## Engulfment Properties of Microglia After Stroke

Whether the phagocytic property of microglia in stroke is harmful or beneficial remains unclear. For instance, some researchers found that upregulating phagocytic capability of microglia in transient middle cerebral artery occlusion (tMCAO) mouse model promoted efficient clearance of tissue debris, facilitated tissue reconstruction, and reorganized neuronal network (24, 25). Conversely, other scholars suggested that excessively activated microglia engulfed live or stressed neuronal cells in endothelin-1 induced focal cerebral ischemia model, which resulted in function deficits and brain atrophy after stroke (26, 27). Therefore, microglial phagocytosis after stroke is a very interesting and complicated biological progress that may depend on the severity of initial ischemia, the advent or not of reperfusion, the location within the lesion (core or penumbra as defined afterwards), and the considered time points post-ictus. In addition, different experimental techniques and stroke models could be one reason for result discrepancy. Therefore, we summarized the various techniques used to measure phagocytic capability in **Table 1**. Another critical reason is that the comprehensive phagocytic effect of microglia may depend on their swallowing different cells mediated by specific “eat-me” signaling pathways. Understanding these mechanisms of microglia-mediated engulfment upon ischemic injury may open up exciting new therapeutic avenues for combating acute-stage inflammation and late-stage synaptic refinements.

**Table 1:** Different techniques available to assess microglial phagocytosis.

Techniques	Concrete experiment content	Advantages/Limitations	Literature
***In Vivo***			
Immunofluorescence (IF): Co-staining with microglial biomarkers, e.g., Iba1	Phagocytosis function: CD68, Lamp1, Phalloidin, Clathrin	The most common method and is suitable for all kinds of phagocytosis, but difficult to show the general view of brain	(28–30)
	Hemocytes: CFSE		(31)
	Neutrophil: NIMP-R14, Ly6G		(31, 32)
	Neuronal cell debris: NeuN, MAP2		(19, 29)
	Endothelial cells: CD31		(33)
	Myelin debris: MBP		(34)
Confocal microscopy and three-dimensional reconstruction	Multiple-immunostaining of Iba1 and other markers followed by orthogonal optical sectioning	Intuitive to show the microglial phagocytosis although the view is small	(27, 33, 32)
Immunohistochemistry	Oil red O staining for myelin debris phagocytosis	Simple and efficient but indirect and non-specific	(35)
Fluorescence activated cell sorting (FCAS)	CD68 expression	A quantitative and overall analysis while nonintuitive	(33)
	Double-positive cells (CD11b and other markers) expression		(32)
Two-photon microscopy	Use transgenic mice visualizing microglia and other cells	Display dynamic phagocytosis process *in vivo* with high technical requirement	(33, 36)
Morphological phenotype: combined with IF	Ball-and-chain microglial buds in developing brain	Only indicating phagocytic activity without definitive evidence	(37)
***In vitro***			
Immunofluorescence (IF)	Use different kinds of fluorescent microbeads to assess phagocytic activity	Might be the “gold standard” but is restricted to *in vitro* study	(27, 32, 38, 39)
Fluorescence activated cell sorting (FCAS)	DiO-labeled myelin debris and microglia coculture	Easy to quantify but the experimental conditions are not unified	(34)
Time-lapse video microscopy	PMN–microglia coculture	Display the phagocytosis procedure in a video but only *in vitro*	(40)
	Neuron–microglia coculture		(39)

CD11b, cluster of differentiation molecule 11b; CD31, Platelet endothelial cell adhesion molecule; CD68, Cluster of Differentiation 68; CFSE, Carboxyfluorescein succinimidyl ester; Iba1, Ionized calcium binding adaptor molecule 1; Lamp1, Lysosomal-associated membrane protein 1; MAP2, Microtubule Associated Protein 2; MBP, Myelin Basic Protein; NeuN, neuronal nuclei; NIMP-R14, a Ly-6G/Ly-6C antibody; Ly6G, Lymphocyte antigen 6 complex locus G6D.

Stroke triggers a cascade of events leading to rapid neuron and oligodendrocyte injury in the infarcted core (unsalvageable infarcted tissue with rCBF values less than 10 ml/100 g/min), and also in the penumbra area (salvageable infarcted tissue with rCBF values of 10 to 22 ml/100 g/min) (41). Initially, stressed neurons or oligodendrocytes release damage associated molecular patterns (DAMPs), which act as eat-me signals attracting microglial phagocytosis. Subsequently, severe or prolonged ischemic injury produces a large number of cell debris. Efficient clearance of cell debris by microglia could be positive feedback for neurogenesis and initiate stroke recovery. Additionally, microglial phagocytosis may destabilize blood–brain barrier (BBB) integrity by engulfing endothelial cells and regulate the inflammatory response by engulfing polymorphonuclear neutrophil granulocytes (PMNs). We here summarized these biological properties.

### The Engulfment of Neurons

It is beneficial in part that microglia are capable of rapidly clearing dead or dying neurons within hours after stroke (39). Therefore, microglia play a fundamental role in facilitating the reorganization of neuronal circuits and resolving inflammation, especially in the ischemic core (42). Nevertheless, in areas of penumbra, stressed but alive neurons expose an eat-me signal called phospholipid phosphatidylserine (PS) in a reversible manner. Phagocytosis of these stressed but viable neurons is harmful to recovery, causing brain atrophy and motor dysfunction, which is also defined as phagoptosis (26, 27). Communication between microglia and stressed neurons is interesting. Upon ischemia, stressed neurons express TMEM16F, which mediates the exposure of PS. TMEM16F knockdown blocked microglial phagocytosis of viable neurons in the penumbra and improved functional recovery in rat MCAO model (43). On the other hand, to resist phagoptosis, neurons may transfer microRNA-98 to microglia *via* extracellular vesicle secretion to prevent the salvageable neurons from microglial phagocytosis in tMCAO model (44).

### The Cleaning of Myelin

In the research on remyelination of multiple sclerosis, it was found that the proliferation of oligodendrocyte progenitor cells (OPCs) and their migration to the lesion are not pivotal players in remyelination, while the differentiation of OPCs is more critical for remyelination. The debris from injured myelin and death cells suppress differentiation of OPCs (35, 45). Moreover, the mononuclear phagocytic system mediates debris phagocytosis, improves the immune microenvironment of OPCs differentiation, and plays a necessary role in the restoration of white matter damage (34). Although white matter injury occupies nearly half of ischemic infarct volume, few studies are concerned about microglia mediated myelin clearance in ischemic stroke. Pseudoginsenoside-F11 (PF11), an ocotillol-type saponin, exerts neuroprotective effects against ischemic stroke in a way that accelerates the phagocytosis of myelin debris by microglia (38). Mechanically, metabolic analysis showed that the clearance of cholesterol-rich myelin debris by microglia could synthesize desmosterol and activate liver X receptor (LXR) signaling, which resolves inflammation and creates a favorable environment for oligodendrocyte differentiation (28). Whether this mechanism is applicable to stroke model remains to be studied.

### The Cleaning of Cell Debris

Microglia, especially M2 microglia, have the constant ability to clear cell debris in ischemic stroke and consequently attenuate the detrimental effects of inflammation (14, 42). Debris clearance efficiency by microglia could be affected by the local microenvironment. For example, a positive correlation was found between the TNF expression level and phagocytic activity in stroke-lesioned rodent brain (22, 46). The ability of microglia to phagocytize and clean cell debris can be halted by reactive oxygen species (ROS) including superoxide (O2^−^) (47).

In addition to controlling detrimental inflammation, microglial phagocytosis does a remarkable job of promoting neurogenesis through clearing debris. Neurogenesis takes place in regions like cerebral neocortex, subventricular zone (SVZ), and sub-granular zone (SGZ), which happens immediately after brain ischemic challenge (48). Microglia isolated from the SVZ supported neurosphere generation *in vitro*, indicating the supporting role of microglia in neurogenesis in SVZ. Studies demonstrated that prefrontal stroke disturbed homeostasis of microglial phagocytosis, presenting with accumulating apoptotic cells in the SGZ (49). In early postnatal rats subjected to hemisphere ischemia, microglia accumulated and displayed engulfment properties in the ipsilateral SVZ region (37), while in adult rat stroke brain, it is reported that activated microglia increased in ipsilateral SVZ region concomitant with neuroblast migration into the ischemic region (30). Even though knowledge about the effects of microglial phagocytotic populations in SVZ region of ischemia is still scarce, the phagocytic nature of SVZ microglia in adult has been reported (29, 30, 50). Enhancement of phagocytosis by sevoflurane treatment might lead to a favorable microenvironment for neuroblast survival (29, 50). Consistently, intraperitoneal injection of minocycline (a microglial inhibitor) hampered the activation of microglia and obstructed neurogenesis at the same time (29).

### The Engulfment of Polymorphonuclear Neutrophil Granulocytes (PMNs)

PMNs infiltrate the brain parenchyma 1 day after focal ischemia, which induces inflammation and exacerbates neuronal damage through releasing oxygen radicals, proteases, and pro-inflammatory cytokines (51, 52). Thus, the prevention of infiltration and the reduction of the number of PMNs are putatively protective. Applying invading PMNs into organotypic brain slices enhances ischemic neurotoxicity, which could be counteracted by additional application of microglia (31, 53). Several recent studies have shown that microglia can engulf these infiltrating neutrophils (31, 32, 36, 54). In hippocampal slice cultures exposed to oxygen glucose deprivation, microglia engulf apoptotic PMNs and viable, motile, non-apoptotic PMNs, adopting a “chasing behavior” by time-lapse image (40). In the rat ischemic brain, the number of neutrophils is controlled by microglial phagocytosis (31). Two-photon microscopy intuitively presented neutrophils which infiltrated into the ischemic brain parenchyma cross talked with microglia and were engulfed by them. The engulfment of PMNs may prevent the release of neurotoxic compounds because dying PMNs secrete toxic compounds and also living PMNs (31). As such, scavenging PMNs alleviate neuron injury by converting the microenvironment from pro-inflammatory to anti-inflammatory (53).

### The Engulfment of Vascular Endothelial Cells

Using two-photon microscopy and CX3CR1^+^/^GFP^ mice, microglia were detected to expand toward adjacent blood vessels within 24 h post tMCAO (33). Subsequently, these perivascular microglia started to eat up endothelial cells by phagocytosis, which caused the disintegration of blood vessels with an eventual breakdown of the BBB. Accordingly, loss-of-microglia-function studies displayed a reduction in the extravasation of contrast agent into the brain penumbra and a decreased infarct size as measured by MRI.

**Figure 1:**
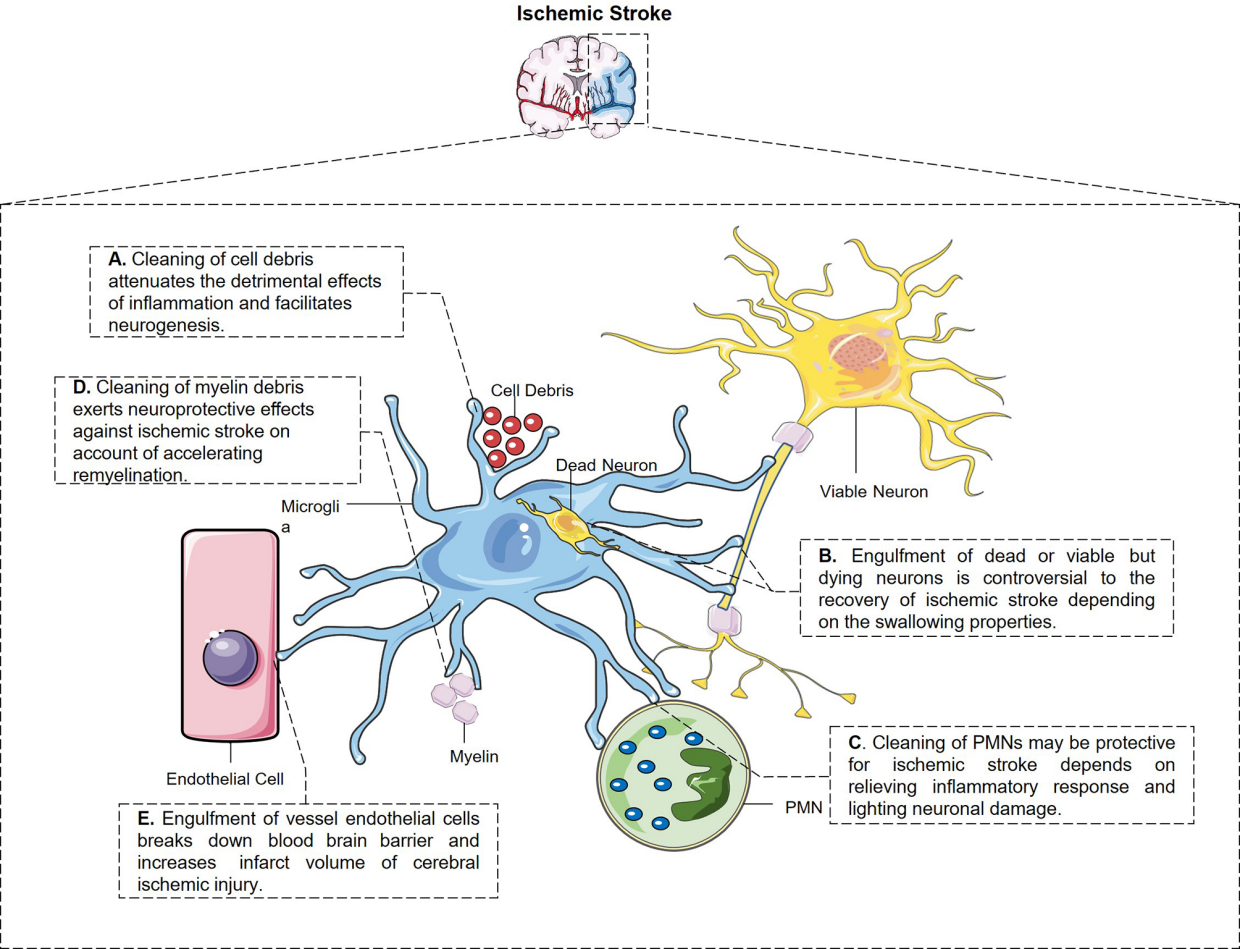
The engulfment properties of microglia and its potential pathological outcome after ischemic stroke. **(A)** Microglia have the constant ability to clear cell debris, which attenuates the detrimental effects of inflammation and facilitates neurogenesis. **(B)** Microglia engulf dead and also stressed but viable neurons after stroke through different signal pathways, which is controversial to the stroke outcome. **(C)** Microglia swallow not only living but also dying PMNs and relieves inflammatory response and neuronal damage from every aspect. **(D)** Microglia swallow myelin debris in MCAO mice which may be protective for ischemic stroke in a way of accelerating remyelination and restoring white matter damage. **(E)** Perivascular microglia eat up endothelial cells after cerebral ischemic injury which breaks down blood–brain barrier and enlarged infarct volume.

Engulfment properties of microglia and their potential pathological outcome after ischemic stroke are depicted in **Figure 1**.

## Signaling Molecules Responsible for Microglia Phagocytosis After Stroke

**Figure 2:**
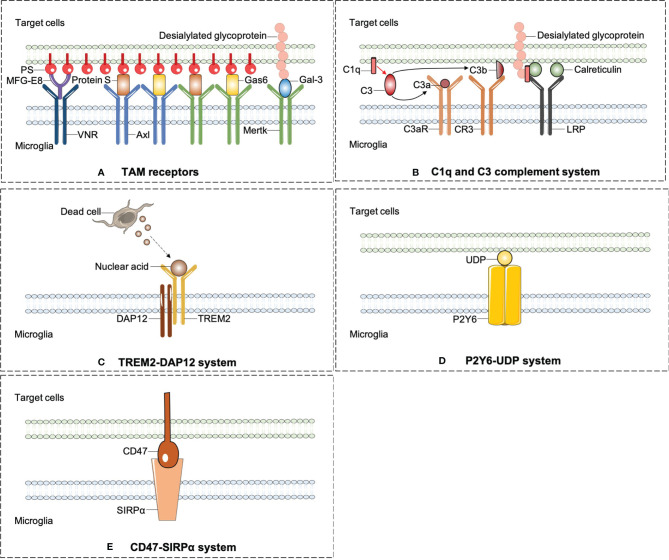
The emergent eat-me/opsonins/phagocytic receptor systems in ischemic stroke. **(A)** Axl and Mertk of TAM receptors were widely expressed on microglia and can identify the best-known “eat-me” signal-PS. PS induces phagocytosis by activating VNR via MFG-E8 or TAM receptors via GAS6 or protein S. In addition to PS, Mertk also binds to desialylated sugar chains of stressed or dying neuronal cells through Gal-3, another opsonin which is rapidly released from activated microglia. **(B)** C1q mediates phagocytic recognition by means of binding to desialylated glycoproteins on targeted cell surface, and cooperatively activates LRP on microglia with the combination of calreticulin. Additionally, C1q triggers a protease cleavage and leads to the deposition of C3b, which directly activates CR3 on microglia. **(C)** TREM2-DAP12 signaling is involved in phagocytosis by microglia and plays a beneficial role in ischemic stroke. **(D)** P2Y6 receptors on microglia is activated by UDP released from damaged neurons and triggers microglial phagocytosis. **(E)** CD47-SIRPαsignaling can inhibit the microglia engulfment property which may aggravate ischemic injury.

Professional phagocytes constantly fulfill a monitoring role rely on immune molecules to identify targeted pathogens or debris in need of removal. All phagocytic behaviors start with the exposure of “eat-me” signals from the target cells or debris. By sensing exposed “eat-me” signals, microglia begin rapid recognition and engulfment of target cells. PS is the best-known “eat-me” signal. Once exposed on the cell surface, it will modulate a crucial process of phagocytosis. PS appears usually inside of the cellular membrane and translocates to the external side of membrane under various circumstances, such as oxidative stress, inflammation, and growth-factor withdrawal (26). Apart from PS, calreticulin and desialylated cell surface glycoproteins also act as “eat-me” signals. Calreticulin is normally localized in the endoplasmic reticulum and translocates on the cell surface as a result of endoplasmic reticulum stress (55). The chemistry process of desialylation is catalyzed by sialidases (also known as neuraminidases). Sialidases integrate on cytomembrane and remove sialic acid residue after cell stress (56). Opsonins are soluble proteins binding the “eat-me” signal to its corresponding receptor on professional phagocytes, which are engaged subsequent to “eat-me” signal exposure. As an illustration, galectin-3 (Gal-3) is one of the opsonins secreted by macrophages and microglia. Upon LPS stimulation, Gal-3 is rapidly released from activated microglia and binds to Mertk on microglial cells and also desialylated sugar chains of stressed or dying neuronal cells (56). Multiple soluble molecules, namely, growth arrest-specific protein 6 (Gas6) or Protein S, milk fat globule EGF factor 8 (MFG-E8) opsonizes PS-exposing cells or other eat-me signals and is subsequently recognized by unique receptors on professional phagocytes (57). Here, we summarized emergent eat-me/opsonin/phagocytic receptor systems in ischemic stroke in **Figure 2**.

### TAM Receptors

TAM is an acronym for Tyro3, Axl, and Mer (protein designation Mer, c-Mer, or Mertk), which is widely expressed on the phagocyte surface of central nervous, immune, vascular, mammalian reproductive system, and retina (57). Mice with triple mutations in *Tyro3*, *Axl*, and *Mer* display a severe lymphoproliferative disorder accompanied by broad-spectrum autoimmunity (58). In the human brain, Axl is highest in mature astrocytes, microglia, oligodendrocytes, endothelial cells, and neurons. Mer expression is highest in both microglia and mature astrocytes, still detected in oligodendrocytes (59). Tyro3 is expressed on mature oligodendrocytes and has been implicated in multiple sclerosis susceptibility and myelin production (60).

Protein S is a vitamin K-dependent anticoagulant plasma glycoprotein. Growth arrest-specific protein 6 (Gas6), a structural analog of protein S, acts as a molecular bridge between the TAM receptors and eat-me signals together with protein S. In retinal pigment epithelial cells, either Mer mutation or concerted deletion of Gas6/protein S ligands disrupts circadian phagocytosis of photoreceptor outer segments (57). As mentioned above, Gal-3 has also been identified as a Mer ligand (61).

The pathological roles of TAM receptors in ischemic stroke have begun to be addressed directly. Protein S blocks endothelial injury and MCAO-induced BBB leakage after ligation of Tyro3 through activating sphingosine 1-phosphate receptor other than Mer or Axl (62). Likewise, protein S obstructs the extrinsic apoptotic cascade through Tyro3-dependent phosphorylation of FKHRL1 which may reduce post-ischemic neuronal toxicity (63). Endogenous expression of Axl and Gas6 increased in microglia/macrophage after stroke, while intranasal injection of recombinant Gas6 (rGas6) reduced the neurological deficits through inhibiting neuroinflammation by inhibiting TLR/TRAF/NF-kappaB pathway (64).

Upon PS exposure, another opsonin molecule MFG-E8 and its microglial receptors vitronectin receptors (VNRs) mediate phagocytosis through activating a CRKII–DOCK180–RAC1 signaling pathway, which results in remodeling of microglial cytoskeleton (26). Previous findings confirmed that microglia express Mer and MFG-E8 in a high-level response to inflammatory stimuli after ischemia stroke. Compared to wild-type mice, mice lacking Mer or MFG-E8 appeared a decreased loss of viable neurons after brain ischemia stroke, which leads to a great improvement of motor function recovery (26, 27). Molecular signaling pathways regarding TAM receptors are summarized in **Figure 2A**.

### C1q and C3 Complement System

The complement components C3 and C1q may induce phagocytosis by binding to dying cell surfaces. C3 is activated and cleaved into the small protein C3a and the larger C3b complex by C3 convertase. C3a could attract immune cells and modulate the immune response by interacting with the cellular receptor C3a receptor (C3aR). C3b along with its cellular receptor CR3 mediates the clearance of dying cells and modulates the adaptive immune response (65, 66).

C1q, the biggest protein of the C1 complex, is present in the neutrophils, microglia, and a subset of interneurons (67). Interestingly, C1q can enhance microglial clearance of apoptotic cells independent of C1r and C1s after ischemic stroke (68). On the intact cell surface, sialic acid modification of glycoproteins or glycolipids can act as a “don’t-eat-me” signal by preventing complement C3b and C1q binding. On the dying cell surface, glycoproteins or glycolipids could be desialylated. Then, C1q mediates phagocytic recognition by means of binding to desialylated glycoproteins. Apart from desialylated glycoprotein, calreticulin also acts as an opsonin for C1q. When C1q opsonizes dead cells, it could not only mediate engulfment by binding to lipoprotein receptor-related protein (LRP) on microglia, but also promote the conversion of C3 to C3b and trigger C3b-based phagocytosis (26, 69, 70).

In the developing CNS, C1q and C3 have been identified as important factors for controlling synaptic pruning. Microglia engulf presynaptic inputs during peak retinogeniculate pruning is dependent on C3b/CR3 signaling pathway (70, 71). After ischemic stroke, complement activation directs continuous microglia-dependent phagocytosis of hippocampal synapses and penumbral salvageable neurons, leading to cognitive decline. B4Crry is a targeted complement inhibitor inhibiting all complement pathways at the central C3 activation step. B4Crry prevents phagocytosis of penumbral neurons and hippocampal synapses, improving long-term motor and cognitive recovery (72, 73). C3aR antagonist, SB 290157 given intracortically, may limit neuroinflammation and neuronal death after ischemia by restraining microglia transition to the phagocytic type (74). In chronic cerebral hypoperfusion rats, SB290157 decreased the number of microglia adhering to myelin, attenuated white matter injury and cognitive deficits (75). Generally, animal studies suggest that activation of the C1q/C3 system after stroke may be detrimental to recovery.

Molecular signaling pathways regarding complement system are summarized in **Figure 2B**.

### Triggering Receptor Expressed on Myeloid Cells-2 (TREM2)-Activating Protein of 12 kDa (DAP12) System

TREM2 was originally described on circulating macrophages, where it is bound to anionic moieties on exogenous pathogens and mediated pathogen clearance (76). DAP12 is an intracellular membrane adaptor of TREM2 (77). Accumulating evidence demonstrated that TREM2-DAP12 signaling is involved in microglial phagocytosis. Human beings with a loss function of either TREM2 or DAP12 develop an inflammatory neurodegenerative disease—Nasu-Hakola disease (NHD), leading to death in the fourth or fifth decade of life (78). TREM2 deficiency in microglia inhibits apoptotic neuronal clearance and increases the production of inflammatory mediators such as TNF-α (79).

TREM2 participates in phagocytic activity following experimental stroke (24, 76). TREM2 deficiency can lead to worsened outcomes after ischemic stroke by decreasing the phagocytosis of injured neurons (24). With the application of bone marrow chimeric mice, this team further addressed that intact microglia TREM2 is more important for beneficial phagocytosis and stroke recovery than that of circulating macrophage (80). Additionally, another research group reported that TREM2 could promote a microglial switch from the detrimental M1 phenotype to the beneficial M2 phenotype, which may affect the short-term outcome in the mouse MCAO model (81). Ligand or opsonin of TREM2 was less reported. It is predicted that the potential endogenous binding partner of TREM2 in ischemic brain is probably high-molecular-weight nucleic acids released by damaged cells (24). In conclusion, the TREM2-DAP12 signaling is mainly considered beneficial in ischemic stroke. Targeting TREM2 signaling especially in microglia has become a therapeutic target as it was shown that the systemic administration of a TREM2 agonist or TREM2 overexpression had a neuroprotective effect in ischemic injury (82).

Molecular signaling pathways regarding TREM2-DAP12 system are summarized in **Figure 2C**.

### Nucleotides and P2 Metabolic Purinoceptors

P2 purinoceptors are divided into two families, ionotropic receptors (P2X) and metabotropic receptors (P2Y). P2Y containing eight types (P2Y1, 2, 4, 6, 11, 12, 13, and 14) are activated by nucleotides and couple to intracellular second-messenger systems through heteromeric G-proteins. P2Y6 receptor is actively responsive to UDP and partially responsive to UTP and ADP. It has been demonstrated that microglial purinergic P2Y6 receptor is activated by UDP released from damaged neurons and triggers microglial phagocytosis (83). In other words, UDP, which is released from injured neurons after trauma or ischemia, acts as an “eat-me” signal and meditates the P2Y6-dependent phagocytosis (53). P2Y6 is combined with UDP, and then activates phospholipase C (PLC) which in turn causes the synthesis of inositol 1,4,5-trisphosphate (InsP3) and triggers the booted release of Ca^2+^ from InsP3-receptor-sensitive stores. On top of triggering the intracellular Ca^2+^ over-loading, the P2Y6-receptor-signaling pathway triggers actin cytoskeleton polarization to shape filopodia-like protrusions, thus facilitating the engulfment of cell debris (83).

The research newly reported that the expression of the P2Y6 receptor in microglia increased after tMCAO and P2Y6 receptor antagonist MRS2578 treatment inhibited microglia to swallow apoptosis cell debris, subsequently aggravating neurological function. The possible mechanism of P2Y6 receptor-mediated phagocytosis is related to myosin light chain kinase (84). After all, it indicates that P2Y6/UDP-mediated microglial phagocytosis plays a favorable part in the acute stage of ischemic stroke, which can be a therapeutic target for ischemic stroke.

Apart from P2Y6/UDP signaling pathway, neuronal injury gives rise to the leakage of ATP or ADP that appears to be a “find-me” signal to attract microglia and cause chemotaxis through P2Y12 receptors (85). In the hippocampus regions of young mice, the percentage of apoptotic cell engulfment significantly reduces in P2Y12 deficient mice, which impacts the neurogenesis of hippocampus SGZ (86). Following cerebrovascular damage, P2Y12-mediated chemotaxis of microglia is central to the maintenance of BBB integrity (87).

The P2Y6/UDP and P2Y12/ADP signaling pathways are depicted in **Figure 2D**.

### CD47-Signal Regulated Protein Alpha (SIRPα) System

Inhibitory signals can also modulate microglial phagocytosis, so-called “don’t-eat-me” signals. In the immune system, SIRPα acts as a “don’t-eat-me” signal, which is required for optimal and appropriate microglial engulfment. CD47 is documented to express on synapses, oligodendrocytes, erythrocytes, and microglia. Its receptor SIRPα is an Ig superfamily protein and has been observed on microglia or macrophage in the CNS (88). The expression pattern of CD47-SIRPα is correlated with peak pruning in the developing retinogeniculate system and prevents excess microglial phagocytosis according to literature (89). This inhibitory engulfment property of CD47-SIRPα hindered the maintenance of myelin integrity following mild brain damage (90). In ischemic stroke, a study using CD47 knockout mice suggests that CD47 deletion reduces brain infarct and swelling at an acute stage in MCAO model through decreasing neuroinflammation (91). Notably, it is interpreted that CD47-deficient erythrocytes are more prone to be cleared by microglia/macrophage. Injection of CD47 knockout blood into mouse brain resulted in quicker clot resolution and less brain swelling than WT blood (92). Consistently, CD47 blocking antibody speeded up hematoma clearance (93) and alleviated atherosclerosis through restoring phagocytosis by microglia/macrophage (94). Exempt from cerebral hemorrhage, these findings provide more clues for the treatment of hemorrhagic transformation in stroke patients. The CD47/SIRPα signaling pathway is depicted in **Figure 2E**.

In addition to the molecules described above, several other phagocytic molecules are still being reported and studied, although their functions in ischemic stroke are unknown. For instance, CD22 blocks microglia-mediated engulfment of myelin debris in the aged brain, while CD22 blockade restores microglial homeostasis and cognitive impairment in aged mice (95).

RXR/PPAR-γ is a critical transcription factor in the nuclear receptor superfamily and is responsible for the expression of scavenger receptors. Mice lacking RXR-α in myeloid phagocytes demonstrated worsened late functional recovery and serious brain atrophy in MCAO dependent model (25).

We still have a long way to thoroughly understand the molecular pathways of phagocytosis. We expect to find specific phagocytosis pathways that protect ischemic brain injury without causing excessive alive neuronal clearance in the future.

## Other Immune Cells Contributing to Phagocytosis in Ischemic Stroke

The lack of precise discriminating markers between the myeloid populations (macrophage and microglia) has led many studies summarized above to generate conclusions based on the grouping of the two populations. In addition to microglia and macrophage, some other cells, namely, astrocytes, peripheral infiltrated PMNs, monocytes, and pericytes have been reported to present engulfment properties as well (22, 54, 87, 96–107).

It was reported that monocyte infiltration occurs as early as 4 h after stroke. Infiltrating monocytes are primarily involved in early debris clearance and have significantly higher phagocytic capacity at 72 h after stroke compared with microglia (22). An earlier study underscored the importance of monocytes to hemorrhage clearance in a model of intracerebral hemorrhage and highlighted their ability to significantly improve long-term outcomes through phagocytic function early after stroke (96). Cell surface scavenger receptor CD36 on monocyte-derived macrophages mediates phagocytosis during the recovery phase in post-stroke brains and plays a reparative role during the resolution of inflammation in ischemic stroke (97).

PMNs elicit rapid immune responses compared with mononuclear phagocytes. The infiltration of neutrophils can be detected within 3 h after the onset of ischemic stroke (54). One subtype of PMNs named N2 phenotype represents a pro-resolving function by producing anti-inflammatory factors or engulfing cellular debris (98, 99). A further study corroborates that the N2 phenotype facilitates neutrophil clearance by macrophage and does not induce neuronal death after ischemic injury. Meanwhile, skewing neutrophils toward the N2 phenotype before stroke reduced infarct volumes at 1 day after MCAO (100).

As BBB components, pericytes reside in the abluminal side of endothelial cells lining the capillaries in the brain. Pericytes are considered to be multipotent progenitor cells and exhibit microglia-like properties after ischemia insult (101). In response to ischemic insult, pericytes could leave blood vessel wall and migrate into the ischemic brain parenchyma, where they express microglia-specific markers, namely, Iba-1 and gal-3. In human stroke postmortem sections, a portion of gal-3 positive pericytes were found in the peri-infarct area (102). Platelet-derived growth factor receptor-β (PDGFR-β) is a specific marker of pericytes. PDGFR-β^+^ pericytes, isolated from ischemic brain tissue, could differentiate into Iba-1^+^ ameboid-like microglia and own the capability to phagocytose latex beads (103).

It has been recently observed that astrocytes, acting like non-professional phagocytes, can also contribute to the elimination of apoptotic neurons, synapses, and cell debris after transient focal ischemia in mice (87, 104, 105). Astrocytes become phagocytic during the late stage of ischemia, and ABCA1 along with its downstream molecules plays a pivotal role in this process (106). Phagocytic astrocytes have a distinct role from microglia given that phagocytic astrocytes were observed in the ischemic penumbra region in the late phase of stroke, while phagocytic microglia were located in the infarcted core in the early stage of stroke (105). Judging from the spatiotemporal pattern of astrocytic phagocytosis after stroke, it is predicted that astrocytes would be involved in the elimination of debris and synapses, thereby leading to repairing/remodeling of the ischemic penumbra region (106, 107).

Nevertheless, resident microglia, astrocyte, monocyte/macrophage system, peripheral neutrophils, and pericytes may cooperatively modulate the removal of debris and synapse architecture after stroke.

## Concluding Remarks

Microglial phagocytosis is a double-sword to immunoinflammation and stroke recovery. Microglia engulf live neurons and endothelial cells, which results in excessive neuronal death and BBB leakage respectively. On the other hand, microglia restrict inflammatory damage *via* engulfing cell debris, cleaning infiltrating neutrophils, and creating an optimal microenvironment for neurogenesis. Therefore, understanding the molecular mechanisms and modulating selective microglial phagocytosis represent an attractive target with a long therapeutic window after stroke in the future.

## Author Contributions 

JJ and MZ wrote the first draft and all co-authors revised the manuscript. All authors contributed to the article and approved the submitted version.

## Funding

This study was supported by the National Natural Science Foundation of China (81971112, 82130036, 81920208017, 81400971, and 81801147), the Natural Science Foundation of Jiangsu Province (BK20191116), the Jiangsu Province Key Medical Discipline (ZDXKA2016020), the Key Research and Development Program of Jiangsu Province of China (BE2020620). The project is sponsored by the “Young Talent Support Program” for the China Stroke Association from the China Association for Science and Technology.

## Conflict of Interest

The authors declare that the research was conducted in the absence of any commercial or financial relationships that could be construed as a potential conflict of interest.

## Publisher’s Note

All claims expressed in this article are solely those of the authors and do not necessarily represent those of their affiliated organizations, or those of the publisher, the editors and the reviewers. Any product that may be evaluated in this article, or claim that may be made by its manufacturer, is not guaranteed or endorsed by the publisher.
